# Supplementary figures and images for: Direct and indirect RANK and CD40 signaling regulate the maintenance of thymic epithelial cell frequency and properties in the adult thymus
Source: Front Immunol. 2024 Nov 29;15:1500908. doi: 10.3389/fimmu.2024.1500908 (PMC11638669; doi:10.3389/fimmu.2024.1500908)

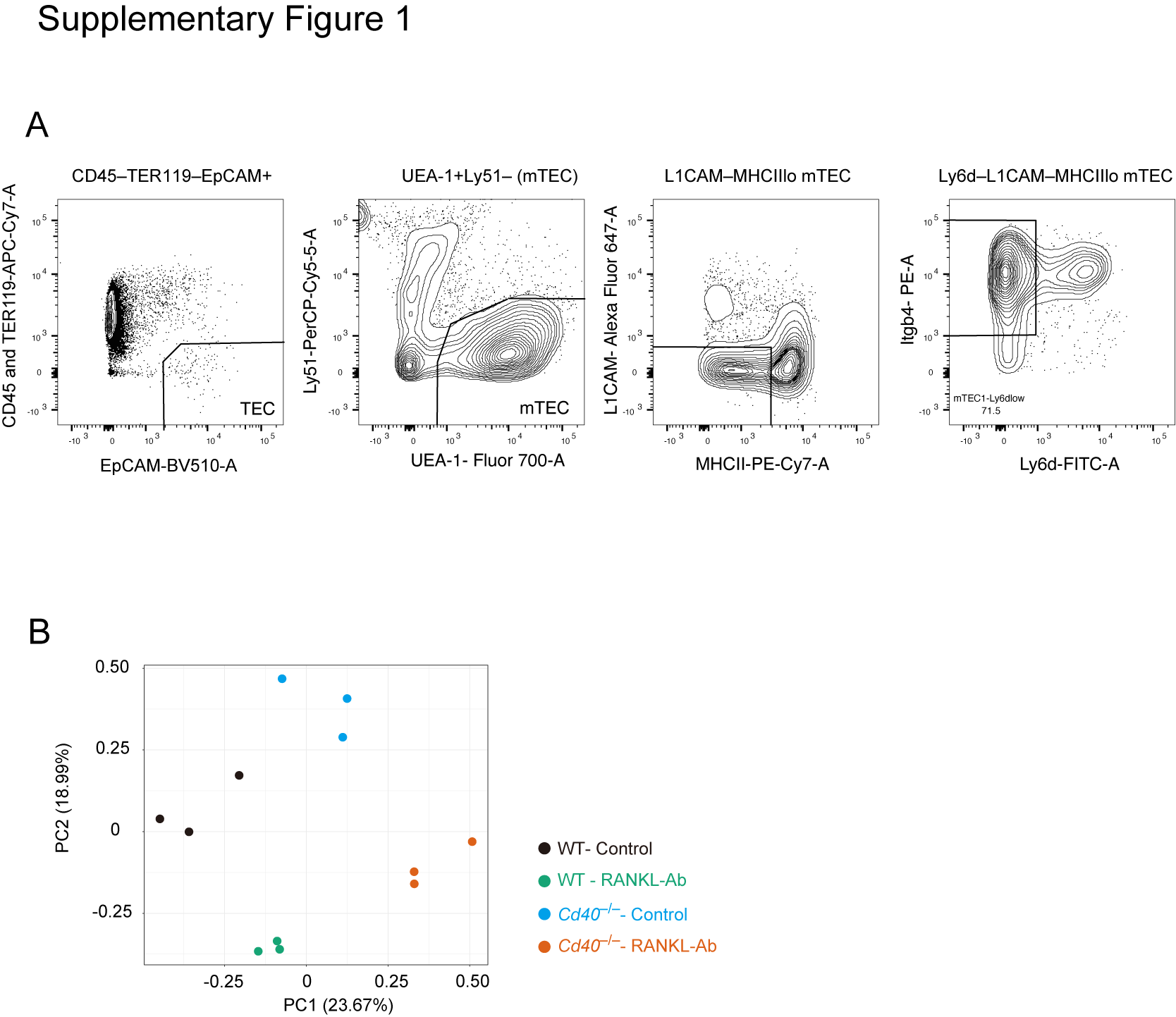

Supplement: Supplementary Figure 1 — Gating strategy and PCA analysis for bulk RNA-seq analysis 1 sis of 2 mTEClo fraction 3 (A) Gating strategy for sorting the mTEClo fraction expressing low levels of L1CAM and Ly6d. 4 (B) PCA analysis of bulk RNA-seq data for the mTEClo fraction. [file Image1.tif]

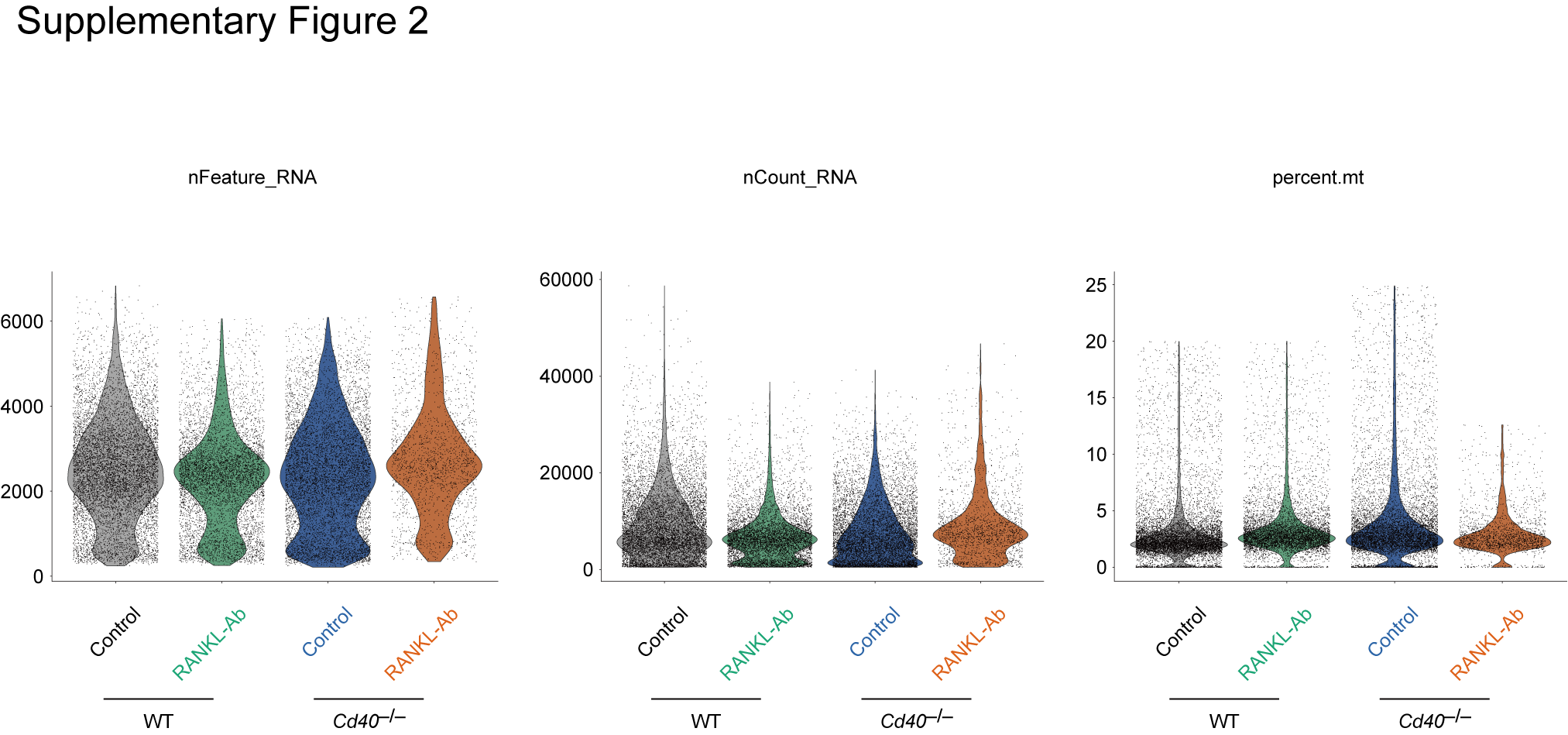

Supplement: Supplementary Figure 2 — Quality control data of single cell RNA-seq data. [file Image2.tif]

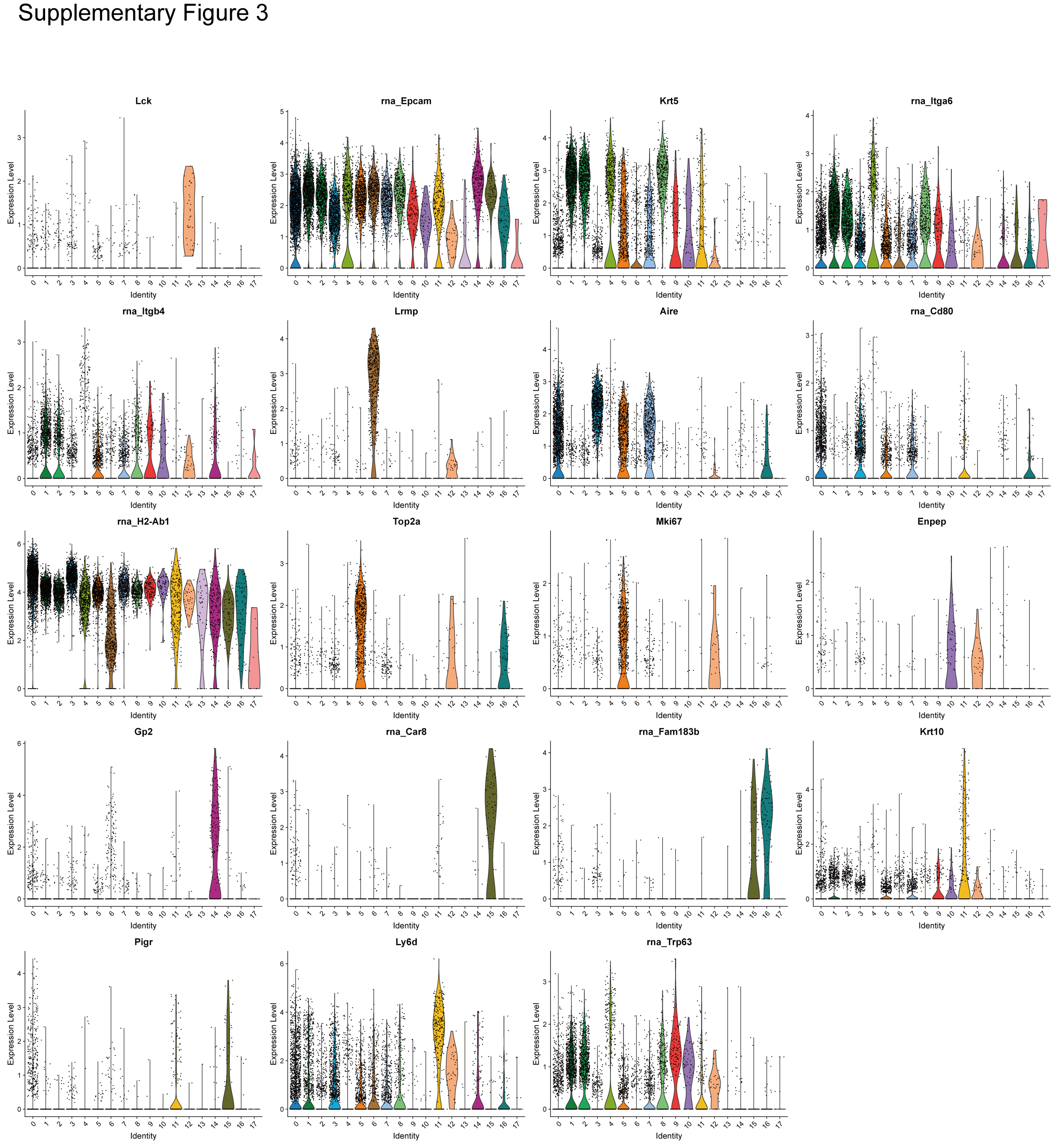

Supplement: Supplementary Figure 3 — Volcano plot of marker gene expression in cell clusters from scRNA9 seq analysis of wild-type mice injected with control IgG. [file Image3.tif]

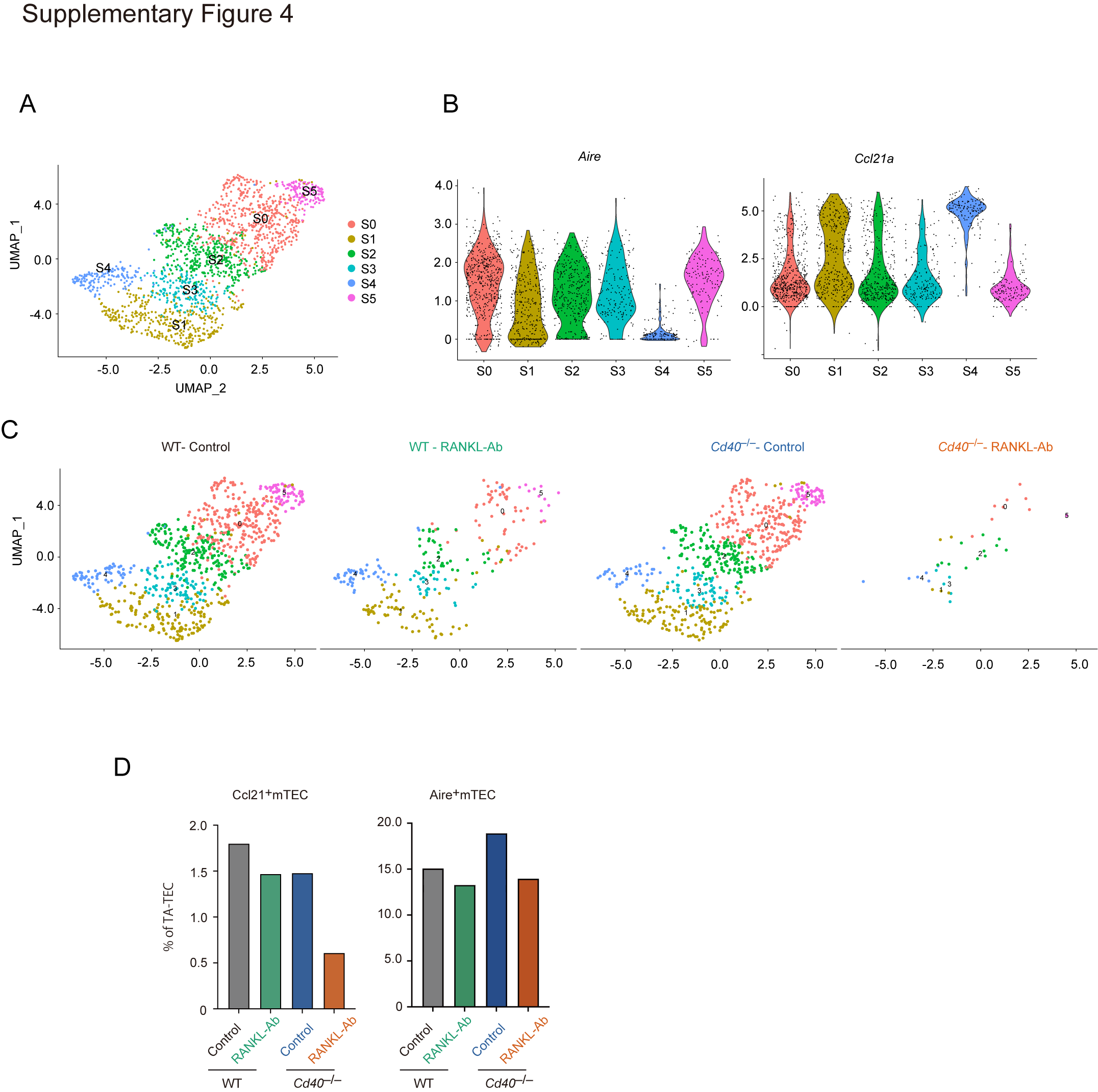

Supplement: Supplementary Figure 4 — Subclustering analysis of TA-TECs. 12 (A) UMAP projection for subclustering of total TA-TECs. 13 (B) Expression of Aire and Ccl21a in each subcluster of TA-TECs. 14 (C) Separation of UMAP projection into individual data sets. 15 (D) Percentages of proliferating cells (TA-TEC subset) in total Ccl21+ mTECs and total Aire+ 16 mTECs. [file Image4.tif]

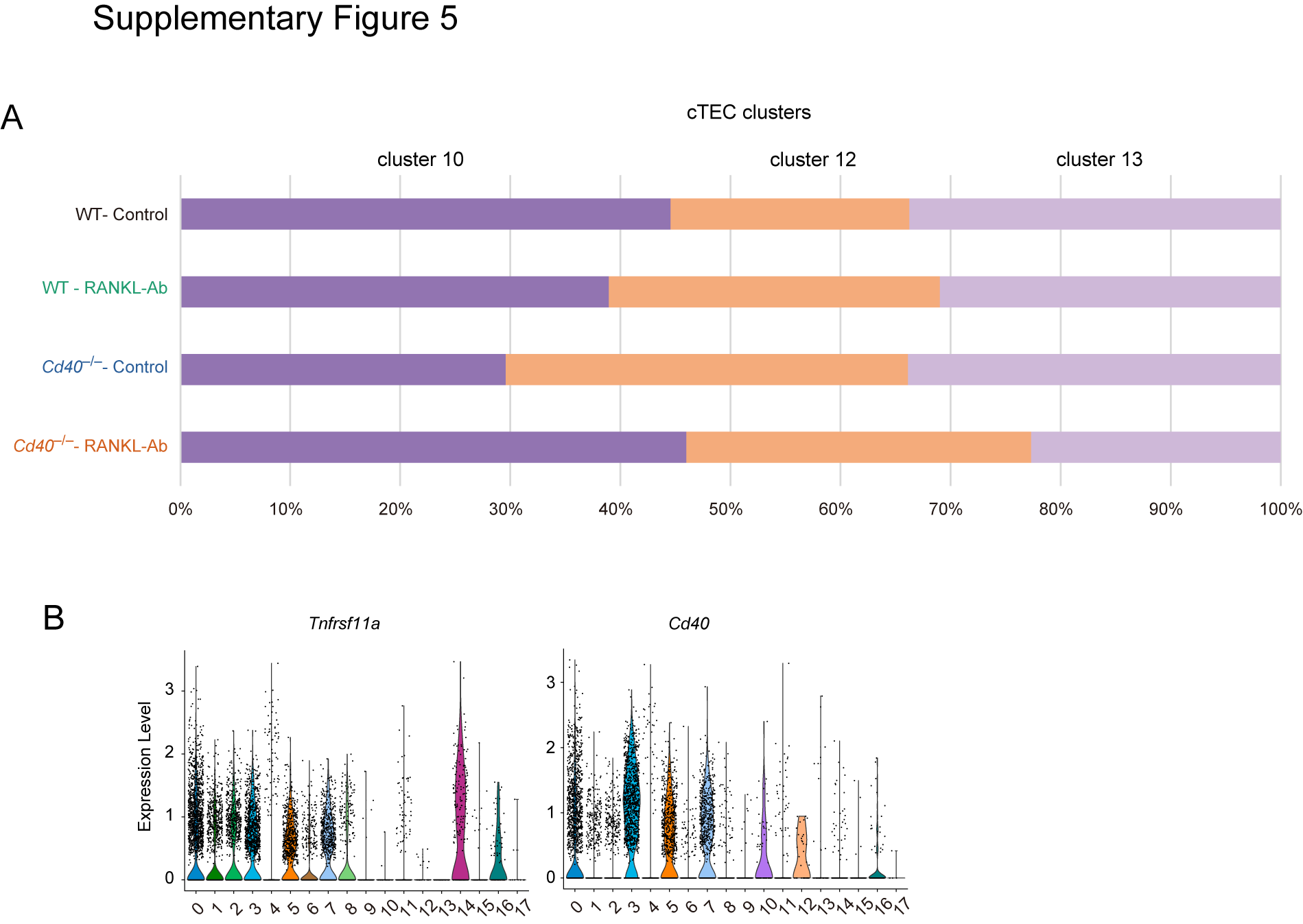

Supplement: Supplementary Figure 5 — Frequency of subclusters in cTEC clusters (A) and expression of 19 RANK and CD40 in TEC clusters from scRNA-seq analysis of wild-type mice injected with 20 control IgG (B). [file Image5.tif]

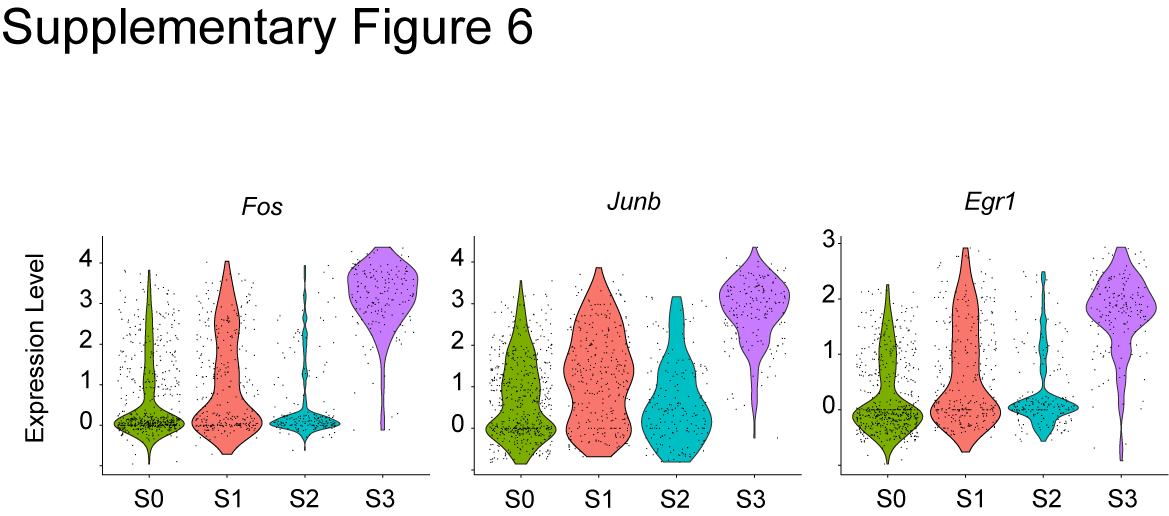

Supplement: Supplementary Figure 6 — Expression of Fos, JunB, and Egr1 in subclusters of TEC progenitors 23 in droplet-based scRNA-seq analysis. [file Image6.tif]

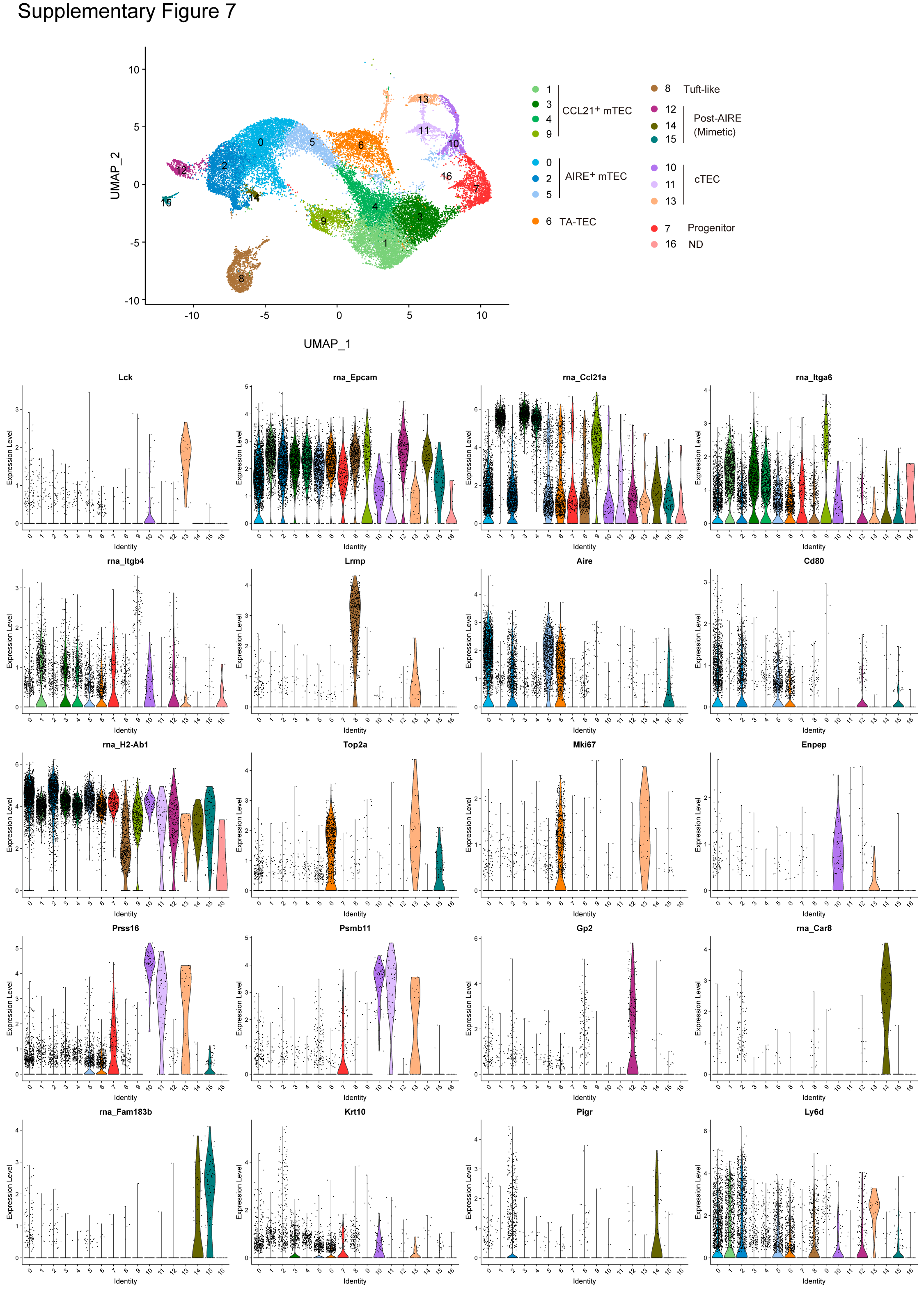

Supplement: Supplementary Figure 7 — UMAP and clustering of integrated scRNA-seq data combining 26 droplet-based scRNA-seq and well-based scRNA-seq data. [file Image7.tif]

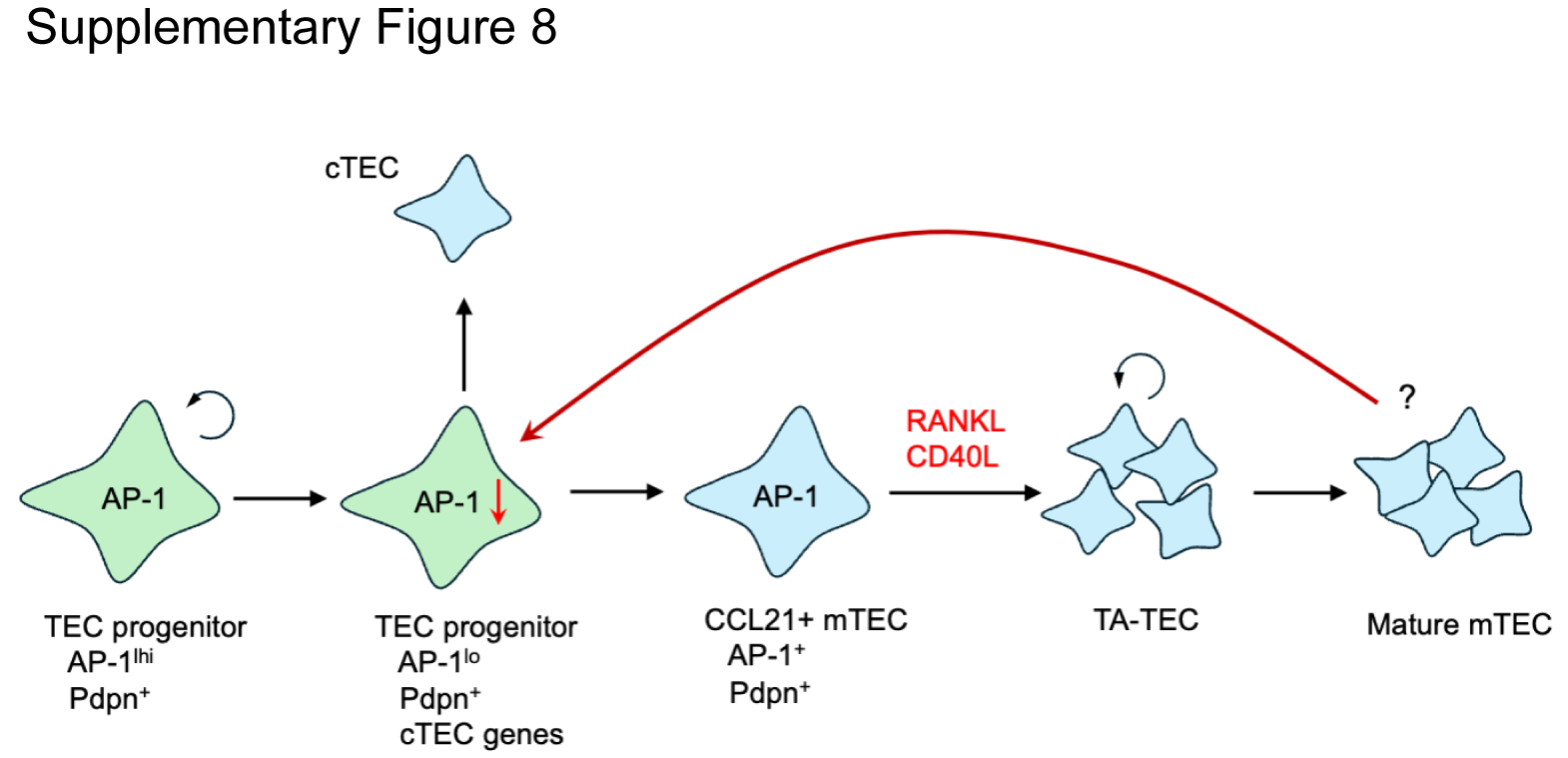

Supplement: Supplementary Figure 8 — A hypothesis for direct and indirect RANK and CD40 signaling in 29 TECs. [file Image8.tif]
